# Prediabetes and cataract risk in adults: A systematic review and meta-analysis

**DOI:** 10.17305/bb.2026.13506

**Published:** 2026-02-05

**Authors:** Linping Xue, Haisong Feng, Beibei Zhang, Dongmei Zuo

**Affiliations:** 1 Hubei University of Chinese Medicine, Wuhan, Hubei Province, China; 2 Department of Neurology, Hubei Provincial Hospital of Traditional Chinese Medicine, Wuhan, China; 3 Department of Integrated Traditional Chinese and Western Medicine, Union Hospital, Tongji Medical College, Huazhong University of Science and Technology, Wuhan, China

**Keywords:** Prediabetes, hyperglycemia, cataract, association, meta-analysis

## Abstract

Prediabetes, characterized by impaired fasting glucose (IFG), impaired glucose tolerance (IGT), or mildly elevated glycated hemoglobin (HbA1c), represents an intermediate metabolic state potentially contributing to cataract formation. However, the existing evidence for this association remains inconsistent. This meta-analysis aims to elucidate the relationship between prediabetes and cataract in adults. A systematic search was conducted across PubMed, Embase, Web of Science, CNKI, and Wanfang for observational studies assessing the association between prediabetes and cataracts in adults. Pooled odds ratios (ORs) and 95% confidence intervals (CIs) were calculated using random-effects models to account for heterogeneity. Subgroup analyses were performed based on study design, geographic region, diagnostic criteria for prediabetes, and covariate adjustment. Eight observational studies involving 22,342 participants were included in the analysis. Of these, 5,305 participants (23.7%) had prediabetes, and 7,625 participants (34.1%) had cataracts. Pooled results indicated that prediabetes was associated with a 34% increased odds of cataract compared to individuals with normoglycemia (OR = 1.34, 95% CI 1.11–1.61; *P* ═ 0.002; I^2^ ═ 50%). Sensitivity analyses restricted to high-quality studies (NOS ≥ 7) produced consistent results (OR = 1.29, 95% CI 1.09–1.53; I^2^ ═ 43%). The association remained significant across subgroups defined by geographic region, mean age, sex, diagnostic criteria for prediabetes, analytical models, and adjustment for sun exposure (all *P* > 0.05 for subgroup differences). In conclusion, this meta-analysis demonstrates a significant association between prediabetes and cataracts in adults. Given that most included studies were cross-sectional, these findings suggest a potential link rather than a causal relationship, highlighting the need for prospective research to clarify temporal relationships and underlying mechanisms.

## Introduction

Cataracts are a predominant cause of visual impairment and blindness globally, accounting for nearly half of all reversible blindness cases, with an estimated 94 million individuals affected [1, 2]. Their prevalence escalates sharply with age, particularly in populations over 60 years, imposing a significant public health burden through reduced quality of life, increased fall risk, and loss of independence [3]. While surgical removal is the only definitive treatment, it demands substantial healthcare resources, particularly in low- and middle-income countries [4]. Thus, identifying modifiable and early-stage risk factors is crucial for developing effective preventive strategies [5]. Various metabolic and systemic factors, including diabetes, hypertension, dyslipidemia, and obesity, have been associated with cataract formation [6]. Among these, hyperglycemia is recognized as a central contributor to lens opacity, primarily through mechanisms such as osmotic stress via the sorbitol pathway, oxidative damage, and non-enzymatic glycation of lens proteins, ultimately leading to structural and functional changes in the crystalline lens [7].

Numerous studies have established a robust association between diabetes mellitus and an elevated risk of cataracts, with both disease duration and glycemic levels significantly influencing this risk [8, 9]. However, the potential impact of prediabetes—a metabolic state that lies between normal glucose tolerance and diabetes [10]—remains less well-defined. Prediabetes includes conditions such as impaired fasting glucose (IFG), impaired glucose tolerance (IGT), or mildly elevated glycated hemoglobin (HbA1c), indicating early glucose metabolism dysregulation without overt hyperglycemia [10, 11]. The global prevalence of prediabetes has surged in recent decades, now affecting over one-third of adults, many of whom progress to type 2 diabetes or develop cardiovascular and microvascular complications [11, 12]. Recent observational studies suggest that even mild elevations in blood glucose may contribute to lens opacity through chronic oxidative stress and protein glycation, although findings have been inconsistent due to variations in study design, population characteristics, and diagnostic criteria [13–20]. To date, no quantitative synthesis has systematically evaluated the association between prediabetes and cataracts within the general population. Therefore, this meta-analysis aims to clarify whether prediabetes is associated with an increased risk of cataracts in adults, integrating evidence from available epidemiological studies and assessing potential sources of heterogeneity.

## Material and methods

This meta-analysis was conducted in accordance with the PRISMA 2020 statement [21] and the Cochrane Handbook for Systematic Reviews and Meta-Analyses [22], addressing protocol development, data collection, statistical procedures, and reporting. The protocol was prospectively registered in PROSPERO (ID: CRD420251207468).

### Literature search

A comprehensive search of PubMed, Embase, Web of Science, Wanfang, and China National Knowledge Infrastructure (CNKI) was performed to identify eligible studies. The search strategy combined the following term groups: (1) (“prediabetes” OR “pre-diabetes” OR “prediabetic” OR “pre-diabetic” OR “borderline diabetes” OR “prediabetic state” OR “impaired fasting glucose” OR “IFG” OR “impaired glucose tolerance” OR “IGT” OR “fasting glucose” OR “HbA1c”); and (2) (“cataract” OR “lens opacity” OR “lens opacities” OR “crystalline opacity” OR “crystalline opacities”). Only full-text, peer-reviewed articles published in English or Chinese and involving human subjects were included. Additionally, references of relevant reviews and original reports were manually checked for further studies. The search encompassed all records from database inception until September 22, 2025. The detailed search strategy for each database is provided in Supplemental File 1.

### Inclusion and exclusion criteria

The selection process for studies was guided by the PICOS framework:

Population (P): Adults (≥ 18 years) from the general population (community-based or health-screening cohorts).

Intervention/exposure (I): Prediabetes, defined by established diagnostic thresholds for IFG, IGT, mildly elevated HbA1c, or their combination.

Comparison (C): Participants with normoglycemia served as the reference group.

Outcomes (O): Incidence or prevalence of cataracts diagnosed according to criteria from the original studies, such as ophthalmologic examination, medical records, or International Classification of Diseases (ICD) codes, compared between subjects with prediabetes and those with normoglycemia.

Study design (S): Observational studies, including prospective or retrospective cohort studies, case-control studies, and cross-sectional studies.

Studies were excluded if they (1) included participants with diagnosed diabetes mellitus without stratified data for the prediabetic group; (2) focused on specific patient populations such as those with ocular trauma, congenital cataracts, or systemic diseases that directly cause cataracts (e.g., corticosteroid-induced, uveitic, or metabolic disorders); (3) lacked a clear definition of prediabetes or normoglycemia; (4) did not provide sufficient data to estimate an effect size (e.g., missing or unadjusted association measures); or (5) were reviews, editorials, conference abstracts, case reports, meta-analyses, or preclinical studies. In cases of overlapping populations, the analysis incorporated the study with the largest sample size.

### Study quality evaluation and data collection

Two investigators independently performed the literature search, screening, quality evaluation, and data extraction, resolving disagreements through consultation with the corresponding author. Study quality was assessed using the Newcastle–Ottawa Scale (NOS) [23], which evaluates cohort selection, control of confounding, and outcome ascertainment. The NOS assigns scores from 1–9, with higher values indicating better quality; studies scoring ≥ 7 were considered high quality. Extracted data included study details (first author, year, design, country), participant information (general characteristics of the population, sample size, mean age, sex distribution), exposure measures (diagnostic criteria for prediabetes and the number of patients with prediabetes at baseline), follow-up duration for cohort studies, outcome definitions (methods for validating the diagnosis of cataracts and the number of subjects with cataracts in each study), and covariates adjusted in the analysis of the association between prediabetes and cataracts.

### Statistical analysis

The association between prediabetes and cataracts in the adult population was quantified by calculating odds ratios (ORs) and 95% confidence intervals (CIs), based on comparisons between participants with prediabetes and those with normoglycemia. When studies reported sex-specific effect estimates for men and women but did not provide an overall (sex-combined) estimate, the male and female strata were treated as independent, non-overlapping datasets, included separately in the primary meta-analysis. Conversely, when both overall and sex-specific estimates were available, only the overall estimate was utilized for the primary pooled analysis, while sex-specific estimates were included exclusively in sex-stratified subgroup analyses. This approach ensured that no study contributed overlapping data to any single analysis while allowing all eligible evidence to be incorporated [22]. When multiple effect estimates were available within a study, the most fully adjusted estimate was preferentially extracted for the primary meta-analysis; crude estimates were used only when adjusted results were unavailable. ORs were employed as the common effect measure for pooling. For prospective cohort studies reporting relative risks (RRs), RRs were converted to ORs using the following approximation based on the baseline risk in the normoglycemic group: OR ═ [RR × (1 -- P0)] / [1 -- (RR × P0)], where P0 represents the prevalence of cataracts in the normoglycemic reference group [24]. Given the moderate effect size and outcome prevalence, this conversion is unlikely to materially influence the pooled estimates. ORs and their standard errors were derived from reported 95% CIs or *P* values and subsequently log-transformed to stabilize variance and normalize the distribution [22]. Between-study heterogeneity was assessed using the Cochrane *Q* test and the I^2^ statistic [25], with thresholds of < 25%, 25%–75%, and > 75% indicating low, moderate, and high heterogeneity, respectively. Pooled estimates were calculated using a random-effects model with the DerSimonian–Laird estimator for between-study variance [22]. Given the limited number of datasets, a sensitivity analysis employing a restricted maximum likelihood (REML) estimator with Hartung–Knapp adjustment was additionally performed to evaluate the robustness of the results [22]. Furthermore, a 95% prediction interval (PI) was calculated for the primary random-effects meta-analysis to estimate the range within which the true effect size of future studies is expected to lie, accounting for both within-study uncertainty and between-study heterogeneity [22]. The PI was derived using the pooled effect estimate and the between-study variance (τ^2^) under the random-effects model [22]. To test robustness, sensitivity analyses were conducted by sequentially omitting individual datasets [26]. Prespecified subgroup analyses further examined whether study-level characteristics influenced the findings, including study country (Asian vs. Western countries), study design (cohort vs. cross-sectional), mean ages of the population (< 65 years vs. ≥ 65 years), sex of the participants, diagnostic criteria for prediabetes, analytic models (univariate vs. multivariate analyses), and adjustment for sun exposure (yes vs. no). These analyses were exploratory, and statistical interaction was assessed using between-subgroup *P* values. Potential publication bias was evaluated through visual inspection of funnel plots and Egger’s regression test [27]. A *P* value < 0.05 was considered statistically significant. All analyses were conducted using RevMan (version 5.3, Cochrane Collaboration, Oxford, UK) and Stata (version 17.0, StataCorp, College Station, TX, USA).

## Results

### Study inclusion

**Figure 1.**
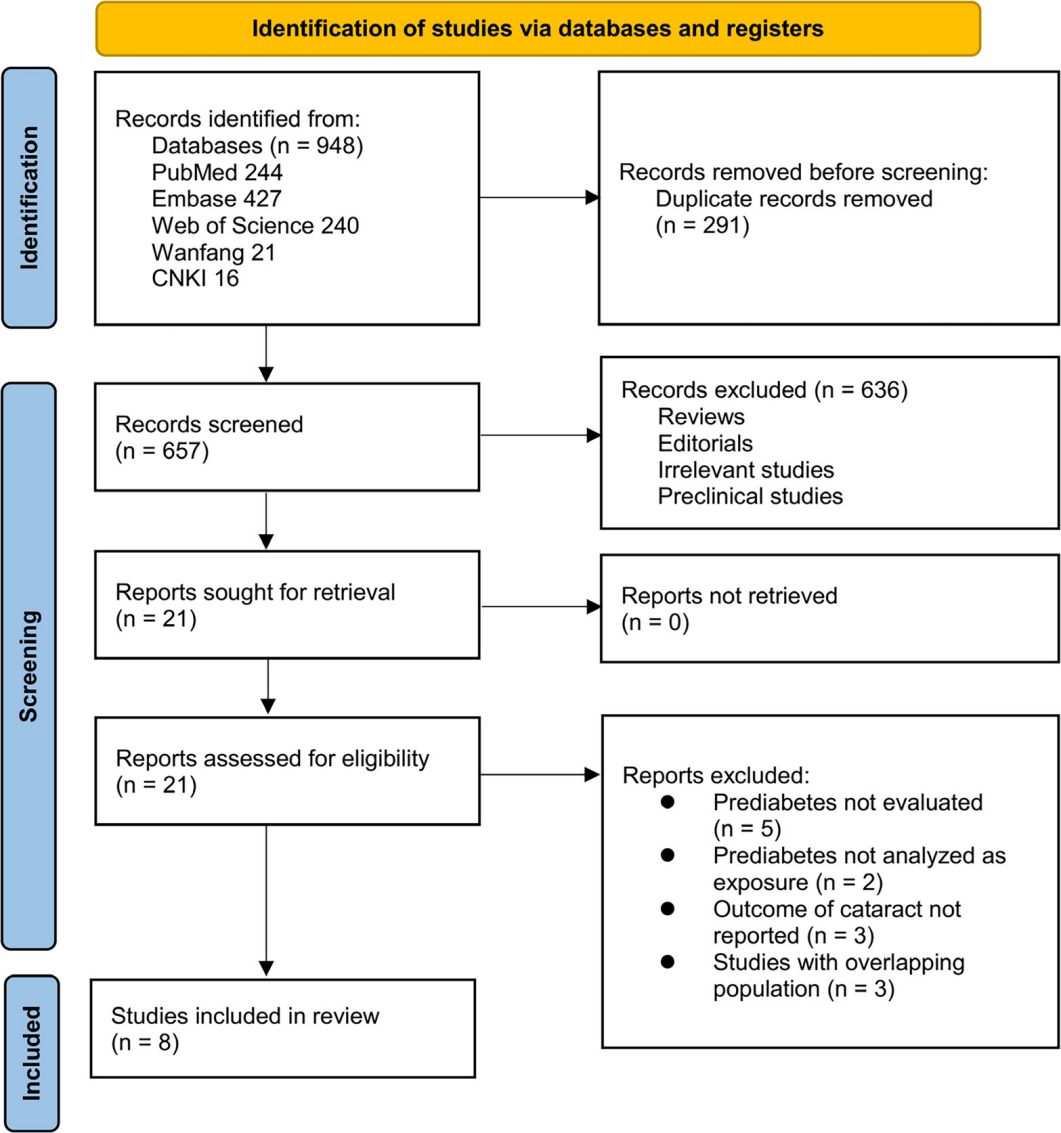
Flow diagram of the study selection process.

The study selection process is illustrated in Figure 1. A total of 948 records were retrieved from five databases, with 291 duplicates removed. Following the screening of titles and abstracts, 636 articles were excluded primarily due to irrelevance to the meta-analysis objective. The remaining 21 full-text papers were independently assessed by two reviewers, leading to the exclusion of 13 studies, as detailed in Figure 1. Consequently, eight studies were ultimately included in the quantitative synthesis [13–20].

### Summarized study characteristics

Table 1 outlines the primary characteristics of the included studies. The analysis incorporated eight observational studies, consisting of seven cross-sectional studies [13–15, 17–20] and one prospective cohort study [16], published between 1984 and 2017. These studies were conducted in Israel, Australia, Lithuania, Singapore, China, South Korea, and Poland, reflecting diverse geographic and ethnic backgrounds. Overall, 22,342 participants underwent community-based eye tests, with the mean age reported to range from 56.7–69.4 years. The proportion of male participants varied from 33.3% to 53.2%. Prediabetes was defined using one or more standard diagnostic criteria, including IFG in five studies [14–16, 18, 19], IGT [13], mildly elevated HbA1c [17], and combined IFG and/or IGT [20], with a total of 5,305 participants (23.7%) diagnosed with prediabetes. The follow-up duration for the cohort study was ten years [16]. Cataract diagnosis was typically based on slit-lamp or retroillumination lens photography, graded using standardized systems such as the Wisconsin Cataract Grading System or the Lens Opacity Classification System III. A total of 7,625 participants (34.1%) were found to have cataracts. Adjustment for potential confounders varied significantly, with five studies accounting for multiple factors, including age, sex, lifestyle habits, comorbidities, and sun exposure to varying extents [14], [16–19], while others provided only unadjusted results [13, 15, 20].

### Study quality evaluation

The quality of the included studies was assessed using the NOS, as summarized in Table 2. The overall NOS scores ranged from 6 to 9, indicating moderate to high methodological quality. The single cohort study [16] achieved a maximum score of 9, reflecting strong representativeness, adequate follow-up, and comprehensive adjustment for confounders. Among the cross-sectional studies, three studies [14, 17, 19] scored 9, and one study [18] scored 8, demonstrating high quality with appropriate adjustment for potential confounding factors. The remaining three studies scored 6 [20] or 7 [13, 15], primarily due to inadequate adjustment for confounders or partial representativeness of cases.

### Meta-analysis results

**Figure 2.**
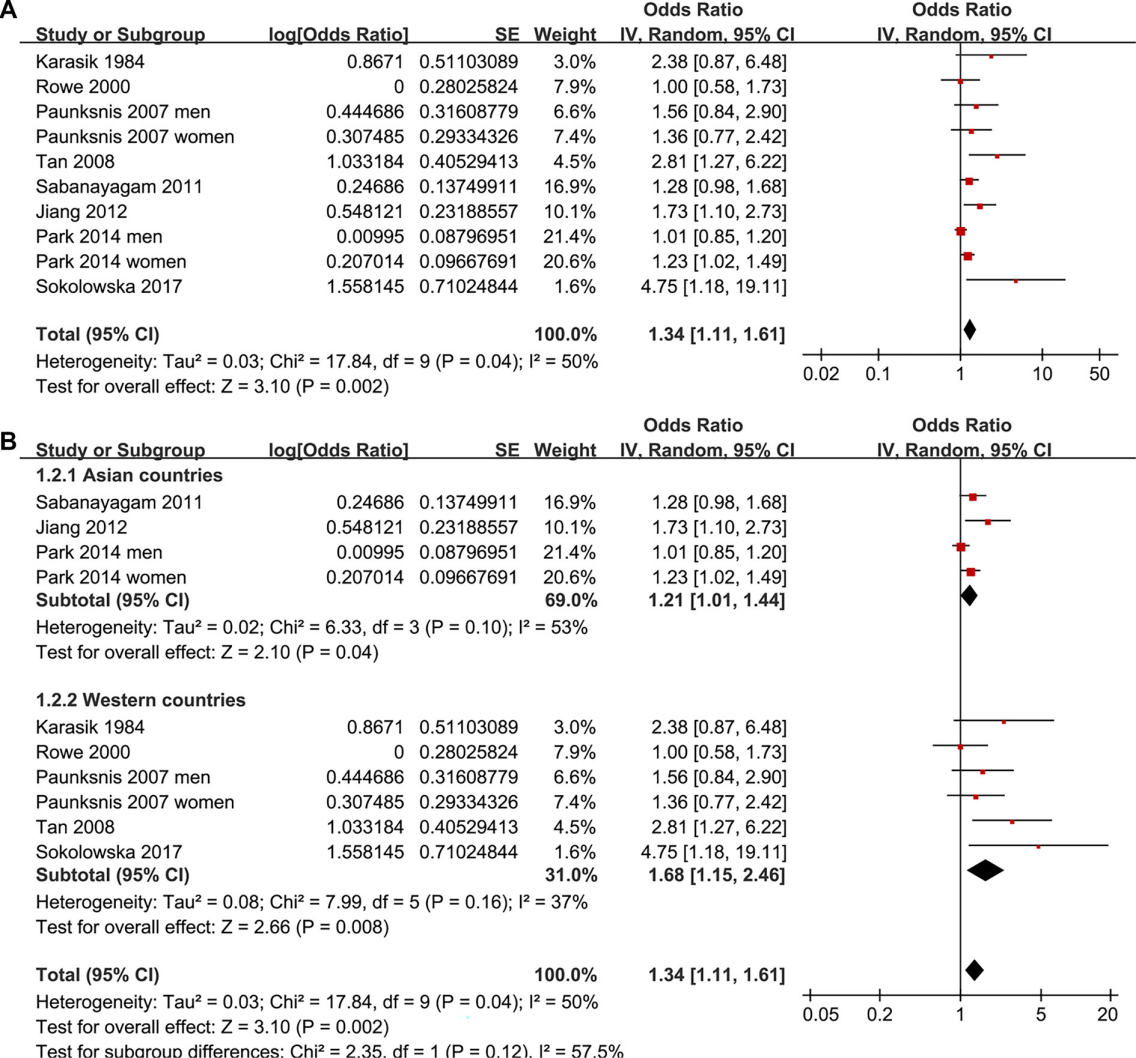
**Forest plots of the association between prediabetes and cataract in adults.** (A) Overall random-effects (inverse-variance) meta-analysis pooling 10 non-overlapping datasets from 8 observational studies comparing prediabetes versus normoglycemia. When studies reported sex-specific estimates, male and female strata were treated as independent datasets and included separately to avoid overlap. (B) Subgroup analysis by study region (Asian vs Western countries). Squares denote study-specific odds ratios (square size proportional to study weight) and horizontal lines indicate 95% confidence intervals; diamonds represent pooled estimates for the overall analysis and each subgroup. The vertical line marks the null value (OR = 1), and the x-axis is displayed on a logarithmic scale. Abbreviations: OR: Odds ratio; CI: Confidence interval.

**Figure 3.**
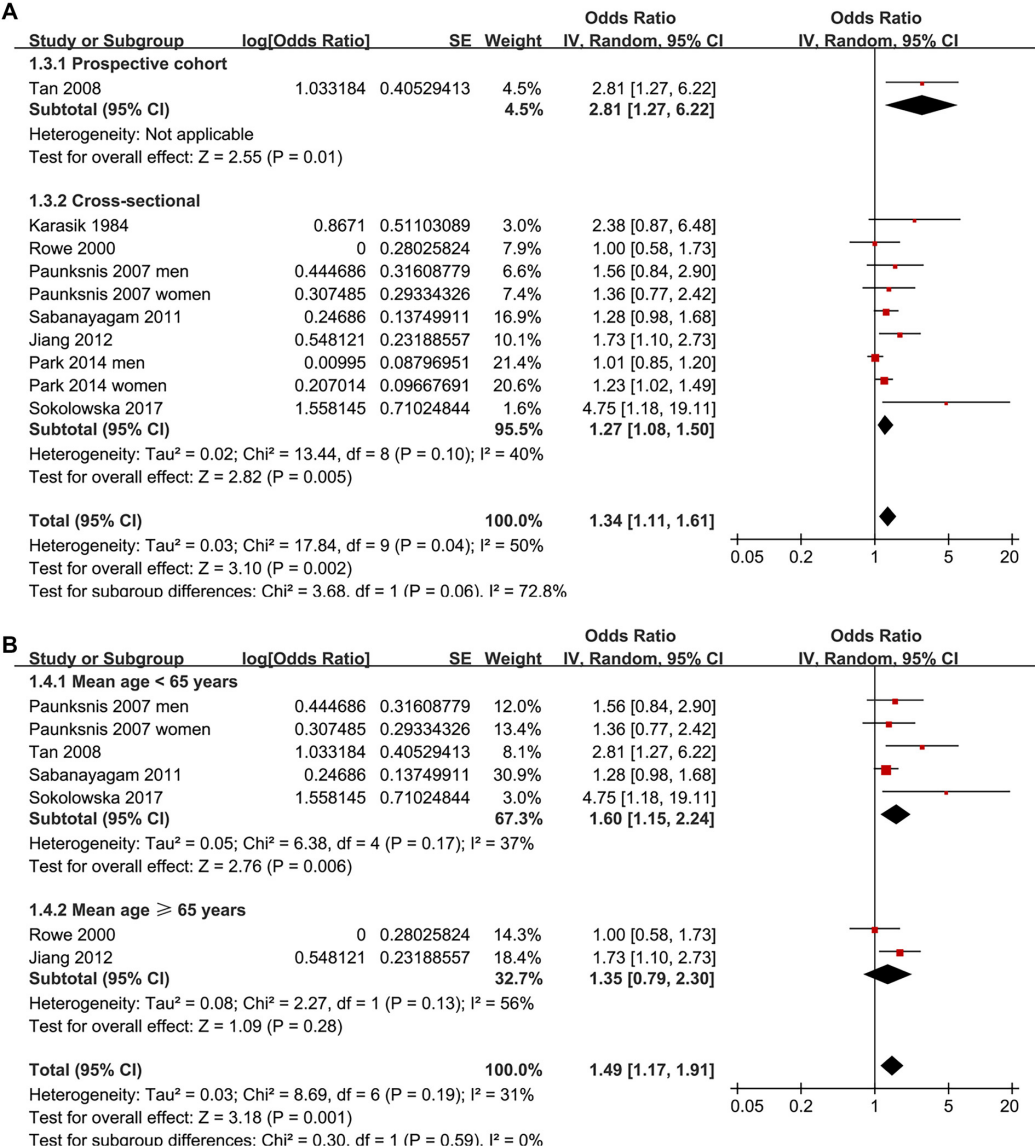
**Forest plots of prespecified subgroup analyses for the association between prediabetes and cataract in adults.** (A) Subgroup analysis by study design (prospective cohort vs cross-sectional studies). (B) Subgroup analysis stratified by the mean age of participants (< 65 vs ≥ 65 years). Study-specific effect estimates are presented as odds ratios with 95% confidence intervals and pooled using a random-effects (inverse-variance) model within each subgroup and overall. Squares indicate individual study estimates (size proportional to study weight) and horizontal lines denote 95% confidence intervals; diamonds represent pooled subgroup and overall estimates. The vertical line indicates the null effect (OR = 1), and the x-axis is on a logarithmic scale. Abbreviations: OR: Odds ratio; CI: Confidence interval.

**Figure 4.**
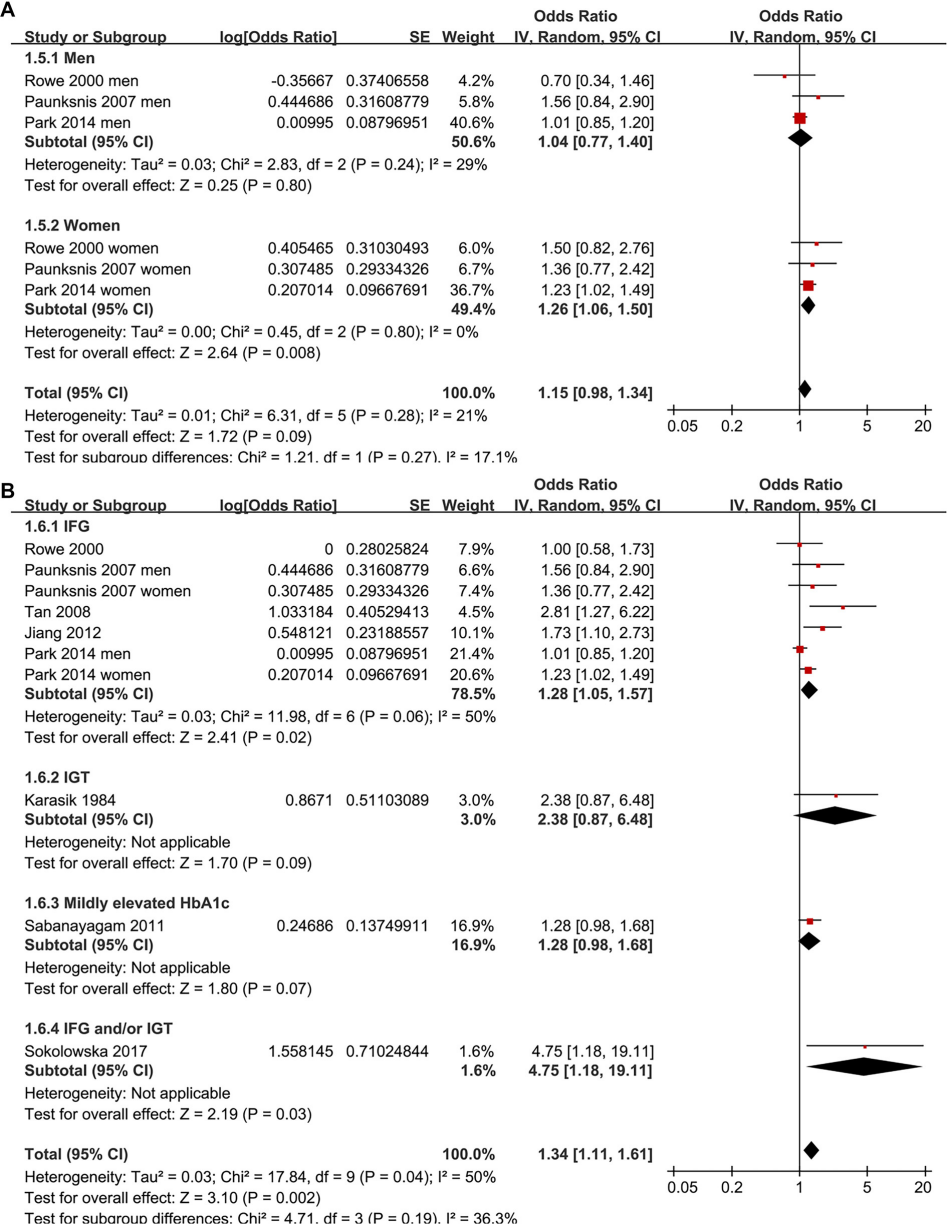
**Forest plots of prespecified subgroup analyses for the association between prediabetes and cataract in adults.** (A) Sex-stratified subgroup analysis (men vs women). Study strata providing sex-specific estimates were pooled within each sex using a random-effects (inverse-variance) model; the χ^2^ test for subgroup differences evaluates effect modification by sex (*P* ═ 0.27). (B) Subgroup analysis according to prediabetes definition, including IFG, IGT, mildly elevated HbA1c, and combined IFG and/or IGT. Random-effects models were fitted within each definition and overall; the χ^2^ test for subgroup differences assesses whether pooled associations differ across diagnostic criteria (*P* ═ 0.19). Squares represent study-specific odds ratios (size proportional to study weight) and horizontal lines indicate 95% confidence intervals; diamonds depict pooled estimates for each subgroup and overall. The vertical line denotes the null effect (OR = 1), and the x-axis is shown on a logarithmic scale. Abbreviations: OR: Odds ratio; CI: Confidence interval; IFG: Impaired fasting glucose; IGT: Impaired glucose tolerance; HbA1c: Glycated hemoglobin.

**Figure 5.**
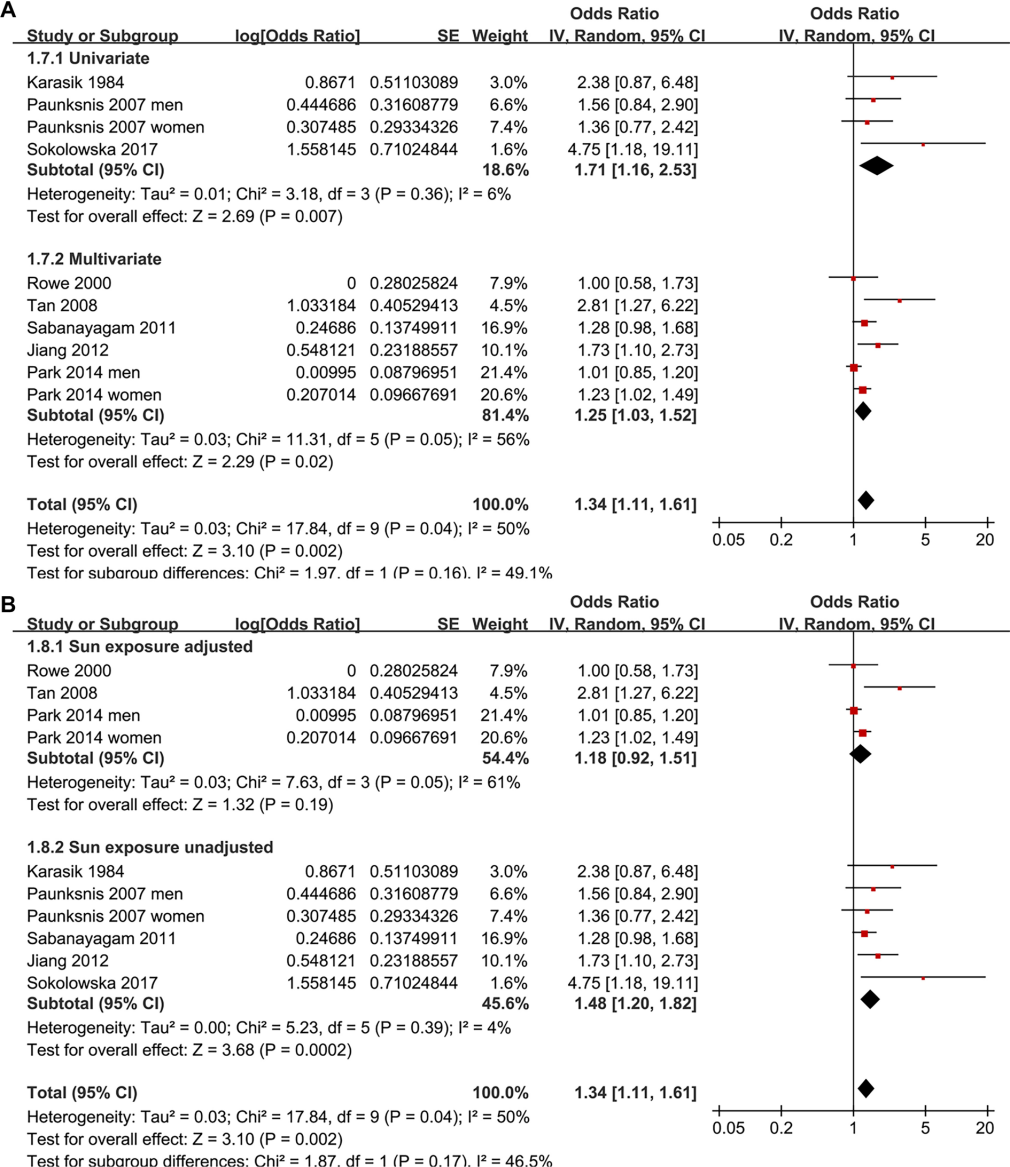
**Forest plots of prespecified subgroup analyses for the association between prediabetes and cataract in adults.** (A) Subgroup analysis by analytic model (univariate vs multivariate estimates). The pooled association appeared numerically stronger in univariate than multivariate analyses (OR = 1.71 vs 1.25; *P* for subgroup difference = 0.16). (B) Subgroup analysis according to whether the original study adjusted for sun exposure (yes vs no). Pooled estimates were similar in studies with versus without sun-exposure adjustment (OR = 1.18 vs 1.48; *P* for subgroup difference = 0.17). Study-specific odds ratios were pooled using a random-effects (inverse-variance) model within each subgroup and overall (overall pooled OR = 1.34, 95% CI 1.11–1.61). Squares represent individual study estimates (size proportional to study weight) and horizontal lines denote 95% confidence intervals; diamonds indicate pooled estimates for each subgroup and overall. The vertical line marks the null association (OR = 1), and the x-axis is displayed on a logarithmic scale. Abbreviations: OR: Odds ratio; CI: Confidence interval.

**Figure S1.**
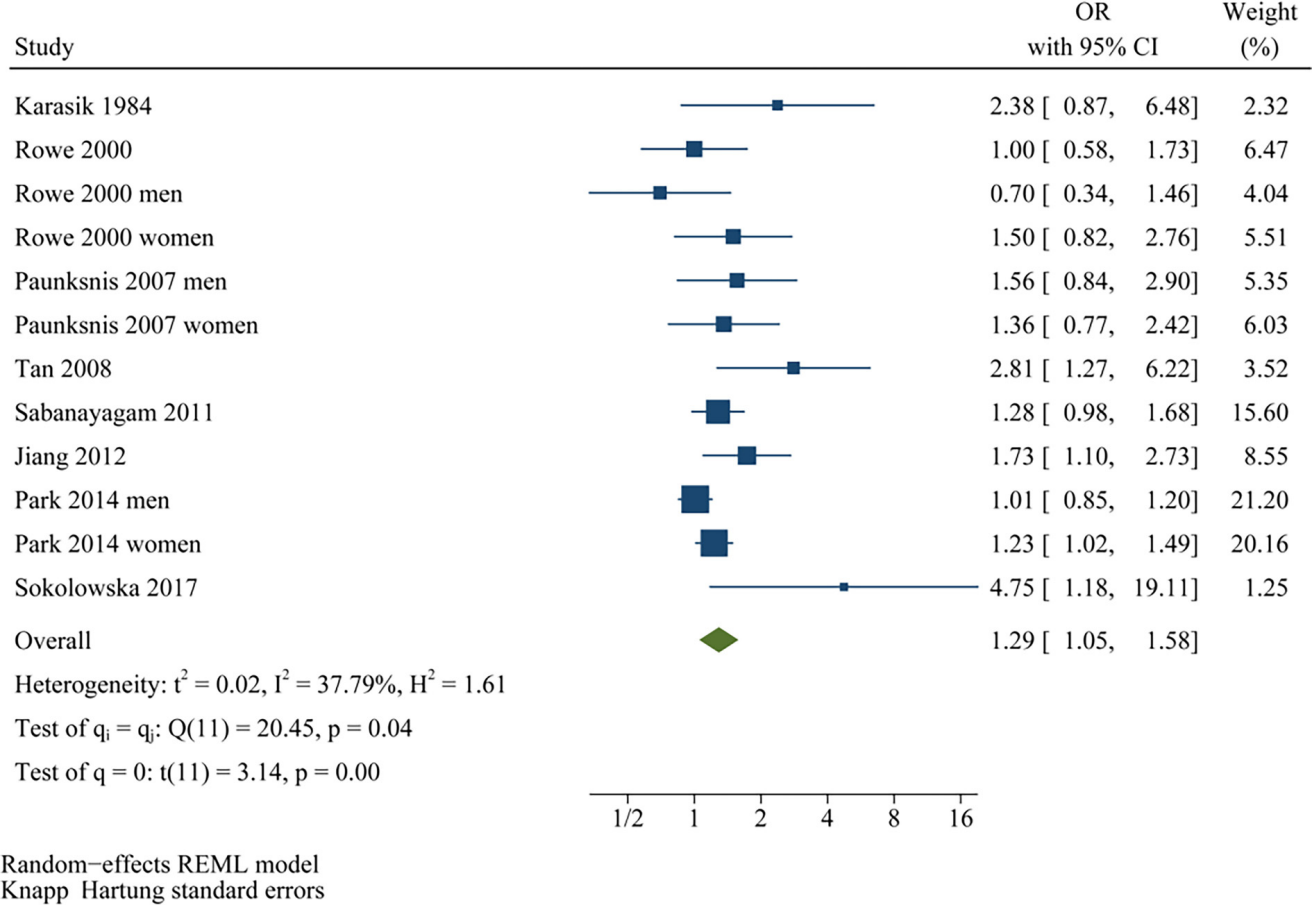
**Sensitivity analysis of the association between prediabetes and cataract using an alternative random-effects specification.** Study-specific odds ratios (squares; size proportional to inverse-variance weight) and 95% confidence intervals (horizontal lines) are pooled using a random-effects model with between-study variance estimated by REML and confidence intervals calculated with the Hartung–Knapp method. The diamond indicates the pooled estimate (OR = 1.29, 95% CI 1.05–1.58), demonstrating results consistent with the primary DerSimonian–Laird analysis and supporting robustness to the choice of random-effects implementation. Between-study heterogeneity is summarized on the plot (I^2^ ═ 37.8%). Abbreviations: OR: Odds ratio; CI: Confidence interval; REML: Restricted maximum likelihood.

Two studies reported associations between prediabetes and cataract in men and women separately [15, 19], leading to their independent inclusion in the meta-analysis. For the study by Rowe et al. (2000) [14], which provided both overall and sex-specific associations, the overall adjusted estimate was included in the primary pooled analysis and all non–sex-stratified analyses, while the sex-specific estimates were exclusively incorporated into the sex-stratified subgroup analysis, ensuring no overlapping data in any single meta-analytic comparison. Pooled results across ten datasets from the eight studies [13–20] demonstrated that prediabetes was associated with an increased risk of cataract in adults compared to those with normoglycemia (OR: 1.34, 95% CI: 1.11–1.61; *P* ═ 0.002; Figure 2A), with moderate heterogeneity (*P* for Cochrane *Q* test = 0.04; I2 = 50%). Sequential exclusion of individual datasets did not significantly alter the findings, with pooled ORs ranging from 1.27–1.42 (all *P* < 0.05). Specifically, sensitivity analysis including only studies of high quality (NOS ≥ 7) [13–19] also yielded consistent results (OR: 1.29, 95% CI: 1.09–1.53; I2 = 43%; *P* ═ 0.003). Additionally, sensitivity analysis using a REML model with Hartung–Knapp adjustment produced similar effect estimates and CIs, indicating that the overall findings were robust to the choice of random-effects implementation (OR: 1.29, 95% CI: 1.05–1.58; I2 = 37.8%, *P* ═ 0.002; Figure S1). The 95% PI for the primary random-effects estimate ranged from OR 0.85–2.11, indicating that while the average association suggests an increased cataract risk, the true effect in any future population could plausibly vary from no association to a substantially elevated risk. Subgroup analyses yielded consistent findings across studies from both Asian and Western countries (OR: 1.21 vs. 1.68, *P* for subgroup difference = 0.12; Figure 2B). The association between prediabetes and cataract appeared stronger in prospective cohort studies than in cross-sectional studies, although the between-subgroup difference was not statistically significant (OR: 2.81 vs. 1.27, *P* for subgroup difference = 0.06; Figure 3A). Further results indicated that the association was not significantly influenced by the mean ages of the populations or the sex of the subjects (*P* for subgroup difference = 0.59 and 0.27, Figure 3B and 4A). Additionally, no significant difference was observed in the association between prediabetes and cataract among studies using varying definitions of prediabetes, including IFG, IGT, mildly elevated HbA1c, and IFG and/or IGT (*P* for subgroup difference = 0.19; Figure 4B). However, results were predominantly influenced by studies defining prediabetes as IFG, necessitating cautious interpretation due to the limited datasets available for other criteria. Similar findings were noted for studies employing univariate and multivariate analyses (OR: 1.71 vs. 1.25, *P* for subgroup difference = 0.16; Figure 5A), and the association between prediabetes and cataract did not significantly differ in studies with or without adjustments for sun exposure (OR: 1.18 vs. 1.48, *P* for subgroup difference = 0.17; Figure 5B).

**Table 1:** Characteristics of the included studies

**Study**	**Design**	**Country**	**Participant characteristics**	**Sample size**	**Mean age (years)**	**Men (%)**	**Criteria for the diagnosis of PreD**	**No. of participants with PreD**	**Follow-up duration (years)**	**Methods for validation of cataract**	**No. of subjects with cataract**	**Variables adjusted**
Karasik 1984	CS	Israel	Nationwide random sample of Jewish population aged 40–70 years	930	NR	53.2	IGT (7.8–11.1 mmol/L)	59	NA	Direct ophthalmoscopy with dilated pupils	57	None
Rowe 2000	CS	Australia	Community population aged 49 years or older	3654	66.0	43.0	IFG (6.1–6.9 mmol/L)	127	NA	Slit-lamp and retroillumination lens photographs, graded by masked assessors using the Wisconsin Cataract Grading System	817	Age, sex, systemic/inhaled steroid use, smoking, alcohol intake, BMI, and sun-related skin damage
Paunksnis 2007	CS	Lithuania	Random sample of the middle-aged (35–64 years) urban population	1282	NR (< 65 years)	44.7	IFG (6.1–6.9 mmol/L)	189	NA	Slit-lamp examination with pupil dilation. Lens opacity graded using the Lens Opacity Classification System III	236	None
Tan 2008	PC	Australia	Community population without cataract at baseline	1796	64.3	43.0	IFG (6.1–6.9 mmol/L)	59	10.0	Slit-lamp and retroillumination lens photographs, graded by masked assessors using the Wisconsin Cataract Grading System	503	Age, sex, and sun-related skin damage
Sabanayagam 2011	CS	Singapore	Community adults aged 40–80 years	2794	58.2	48.3	Mildly elevated HbA1c (6.0–6.5%)	672	NA	Standardized grading from lens photographs (Wisconsin system) or history of cataract surgery	1268	Age, sex, education, and smoking status
Jiang 2012	CS	China	Community adults undergoing eye tests	720	69.4	51.4	IFG (6.1–6.9 mmol/L)	130	NA	Clinical diagnosis, post-operative pathological confirmation of lens opacity.	360	Age, sex, educational level, family residence, economic status, and hypertension
Park 2014	CS	South Korea	Nationally representative sample of non-institutionalized civilians	11076	NR	43.4	IFG (5.6–6.9 mmol/L)	4009	NA	Slit-lamp examination by ophthalmologists, graded according to the Lens Opacities Classification System III	4363	Age, sex, survey year, income, education, residential area, smoking, alcohol, exercise, occupation, family history of eye disease, and sun exposure
Sokolowska 2017	CS	Poland	Adults undergoing eye tests	90	56.7	33.3	IFG (5.6–6.9 mmol/L) and/or IGT (7.8–11.1 mmol/L)	60	NA	Slit-lamp examination (anterior and posterior segment evaluation)	21	None

Studies labeled as “adults undergoing eye tests” were derived from community health screenings or routine eye examinations and were not limited to individuals with diagnosed ocular diseases. For age-stratified subgroup analyses (< 65 years vs. ≥ 65 years), studies with a clearly defined age range were categorized accordingly, even in the absence of a specified mean age. For instance, Paunksnis et al. included participants aged 35–64 years and were thus classified within the < 65 years subgroup. Studies lacking adequate age information were excluded from these age-based subgroup analyses. Abbreviations: CS: Cross-sectional study; PC: Prospective cohort; PreD: Prediabetes; NR: Not reported; NA: Not applicable; IGT: Impaired glucose tolerance; IFG: Impaired fasting glucose; BMI: Body mass index; HbA1c: Glycated hemoglobin.

**Table 2:** Evaluation of study quality using the Newcastle-Ottawa Scale

**Cohort study**	**Representativeness of the exposed cohort**	**Selection of the non-exposed cohort**	**Ascertainment of exposure**	**Outcome not present at baseline**	**Control for age and sex**	**Control for other confounding factors**	**Assessment of outcome**	**Enough long follow-up duration**	**Adequacy of follow-up of cohort**	**Total**
Tan 2008	1	1	1	1	1	1	1	1	1	9
Cross-sectional study	Adequate definition of cases	Representativeness of cases	Selection of controls	Definition of controls	Control for age and sex	Control for other confounders	Exposure ascertainment	Same methods for events ascertainment	Non-response rates	Total
Karasik 1984	1	1	1	1	0	0	1	1	1	7
Rowe 2000	1	1	1	1	1	1	1	1	1	9
Paunksnis 2007	1	1	1	1	0	0	1	1	1	7
Sabanayagam 2011	1	1	1	1	1	1	1	1	1	9
Jiang 2012	1	0	1	1	1	1	1	1	1	8
Park 2014	1	1	1	1	1	1	1	1	1	9
Sokolowska 2017	1	0	1	1	0	0	1	1	1	6

### Publication bias

**Figure 6.**
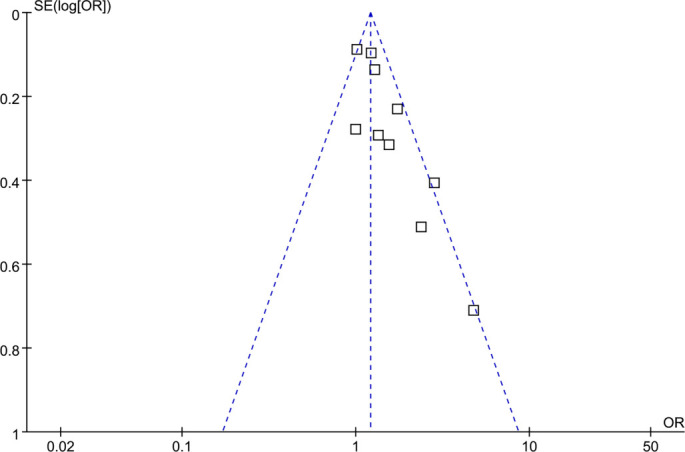
**Funnel plot assessing potential publication bias/small-study effects in the meta-analysis of prediabetes and cataract.** Squares represent individual datasets, plotted as odds ratios (x-axis) against SE[log(OR)] (y-axis). The vertical dashed line indicates the pooled random-effects estimate, and diagonal dashed lines show 95% pseudo–confidence limits. No clear visual asymmetry was observed; Egger’s test was not significant (*P* ═ 0.22). Given the small number of datasets (≈10), these assessments are underpowered. Abbreviations: OR: Odds ratio; SE: Standard error.

As depicted in Figure 6, the funnel plots for the association between prediabetes and cataract did not exhibit significant visual asymmetry. Egger’s regression test did not indicate statistically significant small-study effects (*P* ═ 0.22). However, given the relatively small number of included datasets (approximately ten), these analyses are underpowered, and the presence of publication bias or small-study effects cannot be reliably excluded, even in the absence of apparent funnel plot asymmetry.

## Discussion

This meta-analysis demonstrates that adults with prediabetes have an increased likelihood of developing cataracts compared to those with normoglycemia, and this association remains robust across various analytical approaches. Rather than suggesting a singular pathway or population subgroup driving the findings, the overall pattern supports a consistent, modest excess risk across diverse settings and definitions. These observations expand upon existing evidence regarding hyperglycemia-related lens damage [28], indicating that cataractogenesis may initiate prior to the onset of overt diabetes during the intermediate dysglycemia stage.

Several biologically plausible mechanisms may underlie the observed association between prediabetes and cataract. Even mild, chronic elevations in glucose can activate the polyol (sorbitol) pathway in the lens, leading to osmotic stress, fiber cell swelling, and disruption of lens transparency [29–31]. Low-grade hyperglycemia is also sufficient to enhance oxidative stress and promote the formation of advanced glycation end-products [32], which facilitate cross-linking and denaturation of crystalline proteins [33], thereby altering lens refractive properties [34]. Furthermore, prediabetes often accompanies other metabolic disturbances (such as dyslipidemia, low-grade inflammation, and endothelial dysfunction) that may further impair lens homeostasis and microcirculation [35, 36]. Although current data do not allow for direct testing of these mechanisms, they provide a coherent framework consistent with the observed epidemiological association.

The subgroup and sensitivity analyses offer additional context for interpreting the robustness and generalizability of the findings. The association between prediabetes and cataract was consistently observed across both Asian and Western populations, suggesting that intermediate hyperglycemia may influence cataract risk in diverse environmental and lifestyle contexts. Similar effect estimates across strata of mean age and sex indicate that the association is not confined to a specific demographic group, although age-related lens susceptibility may modulate absolute risk. The numerically stronger association observed in prospective cohort studies compared to cross-sectional studies aligns with a potential temporal relationship, although the limited number of longitudinal datasets precludes definitive conclusions. The persistence of the association in analyses restricted to higher-quality studies and across subgroups defined by diagnostic criteria for prediabetes, analytic models, and adjustments for sun exposure suggests that the findings are not solely attributable to obvious sources of bias or a single operational definition. However, the predominance of studies defining prediabetes via IFG and the limited number of datasets utilizing alternative criteria emphasize the need for cautious interpretation when generalizing across all forms of intermediate hyperglycemia.

This study possesses several strengths. It represents the first meta-analysis synthesizing evidence on the relationship between prediabetes and cataracts, thereby addressing a critical gap in the literature between established data on diabetes and lens opacity and the increasing prevalence of intermediate hyperglycemia in the general population. The literature search was comprehensive and current, incorporating major English and Chinese databases along with predefined inclusion criteria to minimize selection bias. A rigorous quality assessment using the NOS and the implementation of multiple subgroup and sensitivity analyses enhance the credibility of the findings and facilitate the exploration of heterogeneity rather than reliance on a single pooled estimate. The consistent results observed among studies with higher methodological rigor further support the stability of the association.

However, several important limitations must be acknowledged when interpreting these results. Firstly, most included studies were cross-sectional, which precludes the determination of temporal sequence and raises the possibility of reverse causation or bidirectional influences [37]. Consequently, the observed association does not establish that prediabetes precedes and contributes to cataract development; it merely indicates that these conditions frequently coexist. Secondly, moderate between-study heterogeneity was evident, likely reflecting variations in participant demographics, underlying health status, prevalence of metabolic comorbidities, diagnostic criteria for prediabetes, cataract grading methods, and the extent and type of covariate adjustment. These factors could not be fully disentangled due to the limited number of studies and the absence of individual participant data, restricting our ability to conduct more nuanced analyses based on uniform thresholds, cataract subtypes, or duration of dysglycemia. Additionally, although diagnostic thresholds for prediabetes were explicitly extracted and harmonized, heterogeneity in definitions and limited representation of non-IFG criteria constrained further definition-specific sensitivity analyses. Thirdly, while several studies adjusted for major confounders, residual confounding remains a possibility, including unmeasured influences such as dietary patterns, cumulative ultraviolet exposure, socioeconomic status, medication use, and coexisting ocular or systemic conditions [1, 5]. Fourthly, the relatively small number of eligible studies and datasets diminishes the power to detect subtle subgroup differences and limits the precision of publication bias assessments, even though no significant small-study effect was observed. Furthermore, the primary synthesis was conducted on the OR scale, reflecting the reporting practices of most included studies. Although RRs were converted to ORs using established methods, we did not perform a parallel pooled analysis on a single alternative effect-size scale (e.g., log-RR), thus leaving some degree of uncertainty related to metric harmonization. Finally, although all subgroup analyses were prespecified, they were exploratory in nature and based on a limited number of studies, raising the possibility of chance findings due to multiple comparisons. Therefore, subgroup results should be interpreted with caution and not regarded as definitive. Given the observational nature of the evidence, particularly the predominance of cross-sectional studies and the potential for residual confounding, the findings should be viewed as indicative of an association rather than a causal effect.

These limitations carry significant clinical and research implications. Clinically, the current findings should not be interpreted as establishing prediabetes as a confirmed causal risk factor for cataracts or as sufficient grounds for modifying cataract prevention guidelines. Rather, they suggest that lens changes may arise along the continuum of dysglycemia, reinforcing the broader rationale for early detection and comprehensive management of prediabetes to mitigate systemic and potentially ocular complications. For ophthalmologists and primary care physicians, awareness that patients with prediabetes may have an increased likelihood of coexisting or emerging lens opacity could encourage more vigilant inquiry into visual symptoms or opportunistic lens examinations, particularly in older adults or those with clustered metabolic abnormalities, while avoiding over-screening or overstating risk. Future research should prioritize well-designed prospective cohort studies with standardized definitions of prediabetes and cataracts, detailed characterization of glycemic trajectories, and robust adjustment for metabolic and lifestyle confounders to better clarify temporality and dose-response relationships. Studies that integrate biomarkers of oxidative stress, advanced glycation, and polyol pathway activation, as well as those exploring cataract subtypes, may elucidate underlying mechanisms and identify individuals at particularly elevated risk. Individual participant data meta-analyses would further enable the harmonization of diagnostic criteria, stratification by prediabetes phenotype (e.g., IFG, IGT, elevated HbA1c), and evaluation of effect modification by age, sex, ethnicity, and coexisting metabolic risk factors.

## Conclusion

In conclusion, this meta-analysis demonstrates a statistically significant association between prediabetes and cataracts in adults across diverse populations. However, given the observational design of the included studies, residual confounding and reverse causation cannot be excluded, and the findings should not be interpreted as evidence of causality. Future well-designed prospective studies with standardized definitions and uniform outcome assessments are essential to clarify temporal relationships and quantify absolute risk differences in clinically relevant settings.

## Supplemental data

**Supplemental file 1.** Detailed search strategy for each database

**PubMed**

- #1 “Prediabetic State”[Mesh] OR prediabetes[tiab] OR pre-diabetes[tiab] OR prediabetic[tiab] OR pre-diabetic[tiab] OR “borderline diabetes”[tiab] OR “impaired fasting glucose”[tiab] OR “impaired glucose tolerance”[tiab] OR IFG[tiab] OR IGT[tiab] OR “fasting glucose”[tiab] OR HbA1c[tiab]
- #2 “Cataract”[Mesh] OR cataract*[tiab] OR “lens opacity”[tiab] OR “lens opacities”[tiab] OR “crystalline opacity”[tiab] OR “crystalline opacities”[tiab]
- #3 #1 AND #2

**Embase**

- #1 ‘prediabetes’/exp OR ‘prediabetic state’/exp OR prediabetes:ab,ti OR pre-diabetes:ab,ti OR prediabetic:ab,ti OR pre-diabetic:ab,ti OR ‘borderline diabetes’:ab,ti OR ‘impaired fasting glucose’:ab,ti OR ‘impaired glucose tolerance’:ab,ti OR ifg:ab,ti OR igt:ab,ti OR ‘fasting glucose’:ab,ti OR hba1c:ab,ti
- #2 ‘cataract’/exp OR ‘lens opacity’/exp OR cataract*:ab,ti OR ‘lens opacity’:ab,ti OR ‘lens opacities’:ab,ti OR ‘crystalline opacity’:ab,ti OR ‘crystalline opacities’:ab,ti
- #3 #1 AND #2

**Web of Science**

TS ═ ((“prediabetes” OR “pre-diabetes” OR “prediabetic” OR “pre-diabetic” OR “prediabetic state” OR “borderline diabetes” OR “impaired fasting glucose” OR “impaired glucose tolerance” OR “IFG” OR “IGT” OR “fasting glucose” OR “HbA1c”) AND (“cataract” OR “lens opacity” OR “lens opacities” OR “crystalline opacity” OR “crystalline opacities”))

**Wanfang**


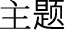
 ═ (“
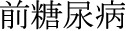
” OR “
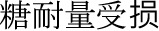
” OR “
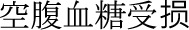
” OR “
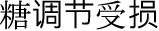
” OR # “
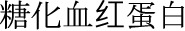
” OR “HbA1c” OR “IFG” OR “IGT” OR “
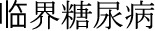
”) AND 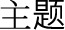 (“
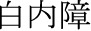
” OR “
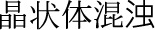
” OR “
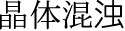
” OR “
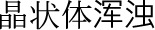
”)

**CNKI (China National Knowledge Infrastructure)**


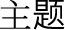
 ═ (“
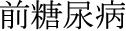
” OR “
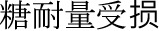
” OR “
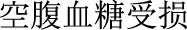
” OR “
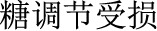
” OR # “
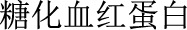
” OR “HbA1c” OR “IFG” OR “IGT” OR “
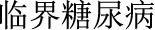
”) AND 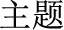 (“
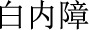
” OR “
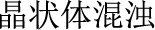
” OR “
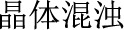
” OR “
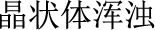
”)

## Data Availability

All data generated or analyzed during this study are included in this published article.
